# Supplementary material for: Effects of a Mediterranean Diet Intervention on Maternal Stress, Well-Being, and Sleep Quality throughout Gestation—The IMPACT-BCN Trial
Source: Nutrients. 2023 May 18;15(10):2362. doi: 10.3390/nu15102362 (PMC10223296; doi:10.3390/nu15102362)
Supplement: Supplementary file 1 [file nutrients-15-02362-s001.zip › nutrients-2381775-supplementary.pdf]

Table S1. Pregnancy and perinatal outcome of women included in the study (n=680).

Characteristics	Usual care<br>n=349	Mediterranean diet<br>n=331	p value
Gestational age at recruitment (weeks)	20.8 (0.7)	20.8 (0.6)	0.64
Pregnancy complications			
Preeclampsia	32 (9.2%)	19 (5.7%)	0.09
Mild	26 (7.5%)	13 (3.9%)	0.05
Severe	6 (1.7%)	6 (1.8%)	0.93
Prenatally diagnosed SGA	31 (8.9%)	18 (5.4%)	0.08
Threatened preterm labor	8 (2.3%)	11 (3.3%)	0.42
Preterm premature rupture of membranes	9 (2.6%)	8 (2.4%)	0.88
Stillbirth	1 (0.3%)	0 (0.0%)	0.33
Delivery outcome			
Gestational age at delivery (weeks)	39.3 (1.9)	39.4 (1.8)	0.21
Preterm birth	20 (5.7%)	22 (6.6%)	0.63
Induction of labor	181 (52.0%)	163 (49.2%)	0.47
Mode of delivery			
Vaginal delivery	204 (58.6%)	174 (52.6%)	0.11
Cesarean section	111 (31.9%)	122 (36.9%)	0.17
Operative vaginal delivery	33 (9.5%)	35 (10.6%)	0.64
Maternal anesthesia[a]	327 (94.2%)	309 (93.4%)	0.63
Antibiotics during labor[b]	157 (45.2%)	166 (50.6%)	0.17
Delivery complications[c,d]	20 (5.8%)	29 (8.9%)	0.12
Neonatal outcome			
Female gender	163 (46.8%)	165 (49.8%)	0.43
Birthweight (g)	3219 (2817-3501)	3250 (2992-3520)	0.12
Birthweight (percentile)	40.8 (30.4)	42.7 (29.1)	0.41
Small for gestational age	75 (21.6%)	46 (13.9%)	0.01
Severe SGA (<3rd centile)	31 (8.9%)	15 (4.5%)	0.02

Apgar 5 minutes <7[e]	1 (0.3%)	1 (0.3%)	0.10
pH umbilical artery[f]	7.2 (0.1)	7.2 (0.1)	0.25
Neonatal resuscitation	17 (4.9%)	12 (3.6%)	0.42
NICU admission	22 (6.3%)	18 (5.4%)	0.63

PE: preeclampsia; SGA: small for gestational age; NICU: Neonatal intensive care unit.
Data are expressed as median (IQR) or mean (SD) or n (%).
[a]Data available for 678 pregnancies.
[b]Data available for 675 pregnancies.
[c]Data available for 674 pregnancies.
[d]Placental abruptio, shoulder dystocia, postpartum hemorrhage, postpartum infection
[e]Data available for 664 pregnancies.
[f]Data available for 459 pregnancies.

Table S2. Changes in dietary key-foods intake and Mediterranean diet adherence evaluated at baseline and final visits according to intervention groups.

		**Mediterranean diet**	**Usual Care**		**Mediterranean diet *vs.* Usual care**
				P[c]	*Difference (95% CI)*
Extra virgin olive oil – g/d	Baseline[a]	34.9 (18.7)	32.5 (19.4)		
	Final[b]	42.6 (0.96)**	39.3 (0.88)**	0.011	3.34 (0.78 to 5.90)
Refined olive oil – g/d	Baseline[a]	7.09 (13.7)	7.94 (15.2)		
	Final[b]	2.98 (0.70)**	5.56 (0.64)*	0.007	-2.57 (-4.43 to -0.72)
Total nuts – g/d	Baseline[a]	21.0 (21.2)	17.8 (18.0)		
	Final[b]	27.5 (1.16)**	23.2 (1.05)**	0.006	4.30 (1.23 to 7.38)
Vegetables – g/d	Baseline[a]	289.8 (129.8)	286.5 (127.8)		

	Final[b]	321.1 (6.40)**	298.4 (5.80)	0.008	22.8 (5.83 to 39.7)
Legumes – g/d	Baseline[a]	52.7 (41.8)	50.8 (34.8)		
	Final[b]	68.4 (2.76)**	63.4 (2.50)**	0.173	5.06 (-2.23 to 12.4)
Fruits – g/d	Baseline[a]	326.2 (169.9)	318.0 (162.2)		
	Final[b]	372.2 (11.2)**	340.8 (10.2)	0.039	31.3 (1.65 to 61.1)
Refined cereals – g/d	Baseline[a]	62.6 (47.3)	63.6 (42.1)		
	Final[b]	37.7 (2.26)**	51.2 (2.07)**	<0.001	-13.5 (-19.5 to -7.45)
Whole grain cereals – g/d	Baseline[a]	41.9 (44.0)	35.4 (36.8)		
	Final[b]	55.7 (2.34)**	46.4 (2.12)**	0.003	9.36 (3.15 to 15.6)
Fish or seafood – g/d	Baseline[a]	72.0 (42.2)	72.4 (43.3)		
	Final[b]	89.6 (2.51)**	78.8 (2.89)*	0.001	10.9 (4.23 to 17.5)
Fat fish – g/d	Baseline[a]	14.9 (16.4)	15.3 (16.1)		
	Final[b]	26.9 (1.20)**	19.7 (1.09)**	<0.001	7.19 (4.01 to 10.4)
Lean meat – g/d	Baseline[a]	71.3 (38.5)	68.9 (37.1)		
	Final[b]	77.8 (2.4)**	72.2 (1.87)	0.043	5.61 (0.18 to 11.0)
Red meat – g/d	Baseline[a]	46.5 (35.0)	50.2 ()36.7		
	Final[b]	42.0 (1.76)*	46.2 (1.61)	0.079	-4.19 (-8.87 to 0.48)
Processed meat – g/d	Baseline[a]	32.0 (27.6)	33.6 (26.1)		
	Final[b]	31.7 (1.20)	30.0 (1.09)*	0.299	1.69 (-1.50 to 4.87)
Pastries, cakes, or sweets – g/d	Baseline[a]	38.0 (32.9)	42.5 (37.5)		
	Final[b]	33.2 (1.86)*	35.7 (1.68)**	0.315	-2.52 (-7.44 to 2.39)
Dairy products – g/d	Baseline[a]	337.5 (214.7)	322.6 (198.5)		
	Final[b]	431.5 (13.0)**	397.6 (11.7)**	0.053	33.9 (-0.38 to 68.2)
Mediterranean diet score	Baseline[a]	7.97 (2.50)	7.46 (2.62)		
	Final[b]	12.1 (0.12)**	7.86 (0.12)*	<0.001	4.26 (3.92 to 4.60)

[a]Baseline values are observed means (SD). [b]Final values are baseline-adjusted (least-squares) means (SE) and comparison among groups done with ANCOVA analysis. *P<0.05 and **P<0.001 final from baseline comparison. [c]ANCOVA analysis.

Table S3. Changes in nutrients intake and Mediterranean diet adherence evaluated at baseline and final visits according to intervention groups.

		**Mediterranean diet**	**Usual Care**		**Mediterranean diet *vs.* Usual care**
				P^c	*Difference (95% CI)*
Energy – kcal/d	Baseline[a]	2468 (520.8)	2420 (509.1)		
	Final[b]	2526 (27.4)*	2502 (24.9)*	0.517	24.0 (-48.6 to 96.6)
Protein – kcal/d	Baseline[a]	103.9 (25.4)	102.7 (26.2)		
	Final[b]	113.6 (1.47)**	108.2 (1.33)**	0.007	5.36 (1.47 to 9.24)
Carbohydrate – g/d	Baseline[a]	222.9 (62.1)	216.0 (58.1)		
	Final[b]	214.5 (3.01)	217.0 (2.74)	0.545	-2.47 (-10.4 to 5.52)
Fiber – g/d	Baseline[a]	33.8 (11.1)	32.9 (10.8)		
	Final[b]	36.4 (0.59)**	34.8 (0.54)*	0.054	1.55 (-0.02 to 3.12)
Total fat – g/d	Baseline[a]	128.8 (30.6)	127.0 (30.0)		
	Final[b]	134.8 (1.65)**	133.4 (1.51)**	0.530	1.41 (-2.98 to 5.79)
SFA – g/d	Baseline[a]	34.7 (10.3)	34.5 (9.41)		
	Final[b]	35.1 (0.53)	35.8 (0.48)*	0.343	-0.68 (-2.08 to 0.73)
MUFA – g/d	Baseline[a]	61.9 (15.0)	61.2 (15.2)		
	Final[b]	64.3 (0.81)*	63.9 (0.73)*	0.685	0.44 (-1.69 to 2.58)
PUFA – g/d	Baseline[a]	22.7 (8.38)	22.0 (7.81)*		
	Final[b]	25.3 (0.48)**	23.8 (0.44)*	0.018	1.53 (0.26 to 2.80)
α-Linoleic acid – g/d	Baseline[a]	14.8 (6.33)	14.5 (5.74)		

	Final[b]	16.6 (0.36)**	15.7 (0.33)*	0.090	0.83 (-0.13 to 1.78)
α-Linolenic acid – g/d	Baseline[a]	1.43 (0.65)	1.38 (0.60)		
	Final[b]	1.96 (0.05)**	1.59 (0.04)**	<0.001	0.37 (0.25 to 0.50)
EPA – g/d	Baseline[a]	0.16 (0.11)	0.16 (0.11)		
	Final[b]	0.23 (0.01)**	0.18 (0.01)**	<0.001	0.04 (0.02 to 0.06)
DHA – g/d	Baseline[a]	0.32 (0.25)	0.33 (0.26)		
	Final[b]	0.50 (0.02)**	0.39 (0.02)**	<0.001	0.10 (0.06 to 0.15)
Trans-FA – g/d	Baseline[a]	1.66 (1.22)	1.66 (1.13)		
	Final[b]	1.31 (0.06)**	1.55 (0.05)	0.003	-0.24 (-0.40 to -0.08)
Cholesterol – mg/d	Baseline[a]	311.8 (98.8)	332.0 (101.4)		
	Final[b]	344.1 (5.25)*	332.8 (4.78)	0.111	11.3 (-2.60 to 25.2)

[a]Baseline values are observed means (SD). [b]Final values are baseline-adjusted (least-squares) means (SE) and comparison among groups done with ANCOVA analysis. *P<0.05 and **P<0.001 final from baseline comparison. [c]ANCOVA analysis.
